# Prognostic value of radiologic and pathological response in colorectal cancer liver metastases upon systemic induction treatment: subgroup analysis of the CAIRO5 trial

**DOI:** 10.1016/j.esmoop.2024.104075

**Published:** 2024-12-11

**Authors:** M.J.G. Bond, C. Mijnals, K. Bolhuis, M.J. van Amerongen, M.R.W. Engelbrecht, J.J. Hermans, K.P. van Lienden, A.M. May, R.-J. Swijnenburg, C.J.A. Punt

**Affiliations:** 1 Julius Center for Health Sciences and Primary Care, University Medical Center Utrecht, Utrecht University, Utrecht, The Netherlands; 2 Department of Pathology, OLVG, Amsterdam, The Netherlands; 3 Department of Gastrointestinal Oncology, The Netherlands Cancer Institute, Amsterdam, The Netherlands; 4 Department of Radiology, Sint Maartenskliniek, Nijmegen, The Netherlands; 5 Department of Radiology and Nuclear Medicine, Amsterdam UMC, Amsterdam, The Netherlands; 6 Department of Radiology, Radboud University Medical Center, Nijmegen, The Netherlands; 7 Department of Radiology, Sint Antonius Hospital, Nieuwegein, The Netherlands; 8 Department of Surgery, Amsterdam UMC, Location University of Amsterdam and Vrije Universiteit Amsterdam, Amsterdam, The Netherlands; 9 Cancer Center Amsterdam, Amsterdam, The Netherlands

**Keywords:** colorectal cancer, liver metastases, pathological response, morphologic response, RECIST

## Abstract

**Background:**

RECIST may not be optimal for assessing treatment response with current systemic regimens. We evaluated RECIST, morphologic, and pathologically documented response (pathological response) in patients with initially unresectable colorectal cancer liver-only metastases (CRLM).

**Patients and methods:**

Four hundred and eighty-nine patients from the phase III CAIRO5 trial were included who were treated with FOLFOX/FOLFIRI/FOLFOXIRI and bevacizumab or panitumumab. The association of the different response tools with overall survival (OS) was evaluated for all patients, and with early recurrence (<6 months) for patients after complete local treatment.

**Results:**

In the overall population, suboptimal [hazard ratio (HR) 1.10, 95% confidence interval (CI) 0.83-1.47] and optimal (HR 0.95, 95% CI 0.74-1.22) morphologic response were not associated with OS compared with no response. RECIST partial response (HR 0.61, 95% CI 0.49-0.76) and progressive disease (HR 5.77, 95% CI 3.97-8.39) were associated with OS compared with stable disease. In 242 patients who underwent local treatment, suboptimal (HR 1.22, 95% CI 0.76-1.96) and optimal (HR 1.28, 95% CI 0.89-1.86) morphologic response were not associated with OS compared with no response. RECIST partial response was not significantly associated with OS (HR 0.73, 95% CI 0.52-1.01), whereas progressive disease was (HR 19.74, 95% CI 5.75-67.78), compared with stable disease. While major pathological response (HR 0.66, 95% CI 0.44-0.99) was associated with OS, partial pathological response (HR 0.82, 95% CI 0.57-1.19) was not, compared with no pathological response. Pathological response, but not morphologic response and RECIST, was significantly associated with early recurrence (*P* < 0.001) which occurred in 13/58 (22%) patients with major response, 29/61 (48%) patients with partial response, and 51/88 (58%) patients with no response.

**Conclusions:**

Our results show that RECIST but not morphologic response was prognostic for OS. In patients eligible for local treatment, neither RECIST nor morphologic response were associated with early recurrence. Pathological response was associated with early recurrence but is only available post-operatively. Hence, novel preoperative parameters are warranted to predict early recurrence and prevent potentially futile liver surgery.

## Highlights

• Different response tools to systemic treatment of CRLM were evaluated.
• Morphologic response was not associated with OS nor early recurrence.
• RECIST response was associated with survival, but not with early recurrence.
• Major pathological response after local treatment was associated with longer survival and less early recurrence.

## Introduction

Radiologic response is an important parameter in the treatment of patients with metastatic colorectal cancer since it determines local and systemic treatment strategies. The standard criteria used to evaluate radiologic response, Response Evaluation Criteria in Solid Tumours (RECIST),^1^ were developed and published in 2000 to assess tumour shrinkage after chemotherapy. However, RECIST may not be optimal for assessing response anymore, as systemic treatment regimens have evolved over the past decade, and targeted agents have been introduced.^2^ In patients with colorectal cancer liver metastases (CRLM) receiving bevacizumab-containing systemic treatment, the computed tomography (CT) morphologic criteria were developed as the surrogate marker for pathologically documented response (pathological response) and overall survival (OS).^3^ Previous studies regarding the prognostic value of morphologic response on OS are conflicting: some were positive,^4^^,^^5^ while others were negative.^6^, ^7^, ^8^, ^9^, ^10^ However, most studies involved small patient populations, had a retrospective design, and rarely used anti-epidermal growth factor receptor (EGFR) antibodies. In addition, these studies have not evaluated the association of the morphologic response criteria with early recurrence in patients undergoing local treatment of CRLM. Early recurrence within 6-8 months after local CRLM treatment is significantly associated with worse OS.^11^, ^12^, ^13^, ^14^ Such an early recurrence after, often extensive, liver surgery may question its efficacy. Prediction of early recurrence could be used to select patients for local treatment, not only to avoid unnecessary exposure of patients to extensive surgeries with its associated perioperative risks, but also to prevent potentially harmful systemic treatment breaks, and reduce health care expenses. Until now, currently available clinical and pathological factors cannot sufficiently predict early recurrence, implying that resectability assessment remains primarily a technical-anatomical decision.^14^^,^^15^

In the multicentre, phase III, randomised CAIRO5 study, patients with initially unresectable CRLM were randomised between combinations of FOLFOX, FOLFIRI, or FOLFOXIRI with bevacizumab or panitumumab, stratified by *RAS*/*BRAF*^*V600E*^ mutation status and sidedness of the primary tumour.^16^ This study aims to investigate the prognostic value of morphologic and pathological response in CRLM upon induction systemic treatment in comparison with RECIST in a prospective cohort of patients with initially unresectable CRLM defined by a central liver expert panel.

## Patients and methods

### Study design and patient selection

Patients with initially unresectable CRLM were included from the phase III randomised controlled CAIRO5 study of the Dutch Colorectal Cancer Group (DCCG) (NCT02162563).^16^, ^17^, ^18^ Patients were randomised between 2014 and 2022 in 46 Dutch centres and 1 Belgian centre. Patients with a right-sided and/or *RAS* or *BRAF*^*V600E*^-mutated tumour were randomised between FOLFOX or FOLFIRI plus bevacizumab and FOLFOXIRI plus bevacizumab. Patients with a left-sided and *RAS* and *BRAF*^*V600E*^ wild-type tumour were randomised between FOLFOX or FOLFIRI plus bevacizumab and FOLFOX or FOLFIRI plus panitumumab.

The study was conducted in accordance with the standards of Good Clinical Practice and the Declaration of Helsinki. The study was approved by the medical ethical committee of the Amsterdam University Medical Centre, Amsterdam, The Netherlands. All patients provided written informed consent to study procedures before enrolment.

### Systemic treatment

Systemic treatment was administered every 14 days for a maximum of 12 cycles or until disease progression, unacceptable toxicity, or patient refusal. If local treatment was planned, bevacizumab was discontinued at least 5 weeks before surgery while chemotherapy could be continued in this period. Adjuvant systemic treatment without the targeted drug (bevacizumab or panitumumab) was recommended to be continued within 12 weeks of (final) local liver treatment to complete the planned 12 cycles. In case of no local treatment, maintenance treatment with fluorouracil and folic acid plus targeted drug was recommended after 12 cycles.

### Resectability assessment by the DCCG liver expert panel

CT scans of patients were evaluated by a central liver expert panel comprising 15 liver surgeons and 3 abdominal radiologists. CRLM were considered unresectable at baseline if an R0 resection could not be done in a single procedure by surgical resection only. Thereafter, patients were reassessed for resectability by the panel every 2 months during systemic treatment allowing all established local treatments (i.e. ablation, two-stage surgery, portal vein embolisation). Each CT scan with panel radiology report was evaluated by three randomly selected panel surgeons, who voted individually on the following categories: resectable, potentially resectable after further induction systemic treatment, or permanently unresectable. If no consensus (i.e. same category selected by all three surgeons) was obtained, two additional surgeons were consulted, and the majority vote was accepted as the final vote. The panel was blinded for the treatment arm.

### Outcomes

Systemic treatment response was based on Response Evaluation Criteria in Solid Tumours version 1.1 (RECIST 1.1).^1^ Radiologic assessment was prospectively carried out by a panel radiologist masked for the assigned treatment. However, for evaluations of CT scans that were done outside the scheduled panel evaluations (i.e. in case of clinical suspicion of disease progression, or when panel evaluations were discontinued when local treatment of CRLM was carried out, or CRLM were considered by the panel as permanently unresectable during systemic treatment), the assessment of the local radiologist was used. Morphologic response was prospectively assessed by a panel radiologist according to the criteria formulated by Chun et al. which divides response into three groups.^3^ Group 1 was characterised by homogeneous low attenuation, with a thin, sharply defined tumour–liver interface, and, if initially present, complete resolution of a peripheral rim of hyperattenuating contrast enhancement. Group 3 was characterised by heterogeneous attenuation, and a thick, poorly defined tumour–liver interface, and a peripheral rim of enhancement may be present. Group 2 was assigned if it could not be classified as 3 or 1. Morphologic response was defined as optimal if metastases changed from group 3 or 2 to group 1, as suboptimal from group 3 to 2, and as no response if the group did not change or increased. In patients with multiple metastases, morphologic response was scored based on the response seen in the majority of metastases. Pathologic characteristics were assessed by an independent pathologist (CM) who was blinded for the treatment arm, for which haematoxylin- and eosin-stained slides were used. Pathological response was scored according to the tumour regression grading (TRG).^19^ The highest TRG score was taken in patients with multiple metastases. Pathological response was also scored according to the mean percentage of viable tumour cells.^20^ Complete response was defined as 0% viable tumour cells, major response as >0%-49%, and minor response as 50%.^3^ The histopathological growth pattern was determined by the pathologist based on the international consensus guideline.^21^ When multiple sections per metastasis or multiple CRLM were present per patient, the mean percentage across sections and lesions, respectively, was calculated.^22^ OS was calculated from the date of randomisation until death or censored on the last clinical visit date. Early recurrence was defined as relapse or death occurring within 6 months after complete local treatment of CRLM.^13^^,^^23^ Death and palliative or no curative-intent local treatment within 6 months after early recurrence were scored as an event for early recurrence without salvage local treatment.

### Statistical analysis

Continuous variables were displayed as medians with interquartile ranges (IQR) and categorical variables as counts and percentages. Median follow-up was estimated using the reverse Kaplan–Meier method. OS was estimated using the Kaplan–Meier method. Hazard ratios (HRs) were estimated with a Cox proportional hazards model and reported with their 95% confidence intervals (CIs). Differences between groups were analysed using Pearson’s chi-square test or Fisher’s exact test, as appropriate. The association between best morphologic and RECIST response with OS was estimated for all patients. Subgroup analyses for best morphologic response were carried out for patients who underwent no local treatment and with RECIST-stable disease. The association of morphologic and RECIST response with pathological response was analysed in patients who underwent local treatment with known pathological response. Pathological response according to the TRG criteria was used in the main analyses, and additional analyses used the percentage of viable tumour cells as pathological response. The association of morphologic, RECIST, and pathological response with early recurrence (≤6 months) and early recurrence without local treatment was analysed in patients who underwent complete local treatment, and with OS in all patients who received local treatment. All analyses were repeated in patients receiving bevacizumab-containing treatment since the morphologic criteria were originally developed for these patients.^3^ An exploratory analysis was carried out to investigate the prognostic value of the collected pathologic characteristics for OS. A two-sided *P* ≤ 0.05 was considered statistically significant. All analyses were carried out in R (version 4.2.2).

## Results

**Figure 1:**
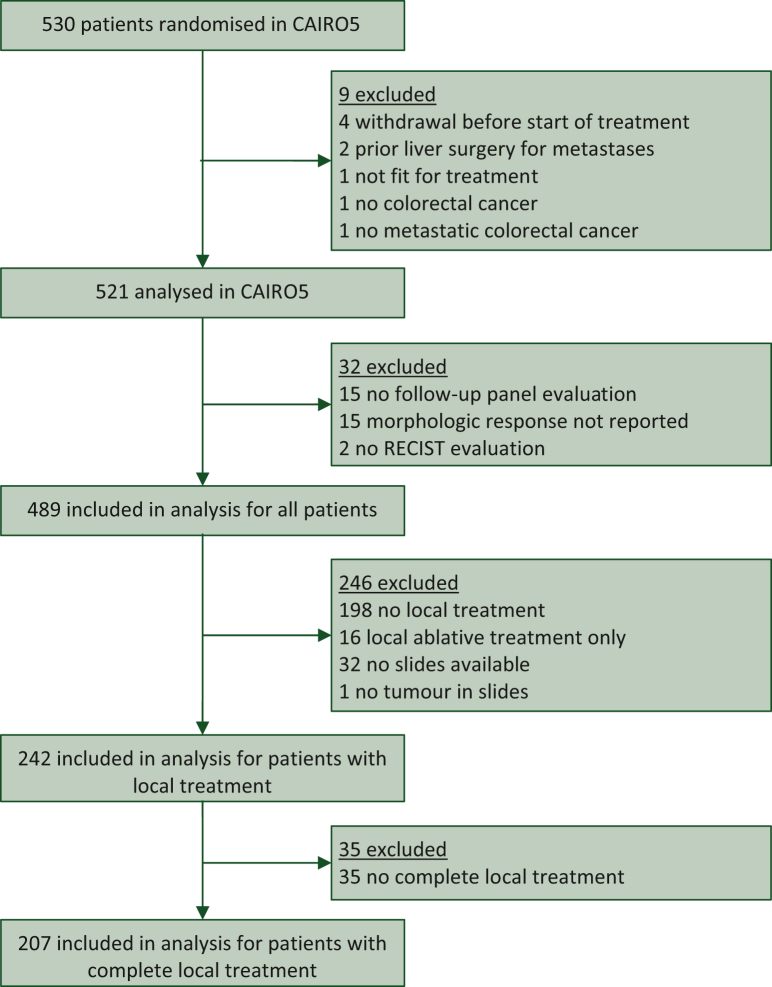
Flowchart of eligible patients.

In total, 530 patients were randomised in CAIRO5 of whom 9 ineligible patients were excluded (Figure 1). Thirty-two patients were excluded for reasons of lack of follow-up by the expert panel, unknown morphologic response, and non-evaluable RECIST response. Pathological response was available in 242 patients who underwent local treatment. Characteristics of all patients are provided in Table 1. The median follow-up was 62.4 months (95% CI 60.5-64.8 months) in the total population. The RECIST response relied on assessment of the panel radiologist in all cases. Early recurrence outcomes were all determined by the local radiologist’s assessment. Morphologic and RECIST response per treatment group are presented in Supplementary Table S1, available at https://doi.org/10.1016/j.esmoop.2024.104075. Morphologic response was not significantly different between treatment groups among patients with a right-sided and/or *RAS* or *BRAF*^*V600E*^-mutated tumour (*P* = 0.21), nor among patients with a left-sided and *RAS* and *BRAF*^*V600E*^ wild-type tumour (*P* = 0.086).

**Table 1:** Patient characteristics

	Overall (*N* = 489)
Age (median, IQR)	62 (54-69)
Sex	
Female	185 (38%)
Male	304 (62%)
Performance status	
0	316 (65%)
1	170 (35%)
2	2 (0%)
Missing	1 (0.2%)
Primary tumour sidedness	
Left	363 (74%)
Right	126 (26%)
Time to metastases	
Metachronous	51 (10%)
Synchronous	438 (90%)
Prior adjuvant chemotherapy for primary tumour	
No	468 (96%)
Yes	21 (4%)
Prior radiotherapy for primary tumour	
No	423 (87%)
Yes	66 (13%)
*RAS* mutation	
No	253 (52%)
Yes	236 (48%)
*BRAF*^*V600E*^ mutation	
No	460 (94%)
Yes	29 (6%)
Total number of liver metastases (median, IQR)	12 (7-22)
Size of the largest liver metastasis (median, IQR)	41 (27-66)
Systemic treatment	
FOLFIRI plus bevacizumab	22 (4%)
FOLFIRI plus panitumumab	12 (2%)
FOLFOX plus bevacizumab	226 (46%)
FOLFOX plus panitumumab	97 (20%)
FOLFOXIRI plus bevacizumab	132 (27%)

IQR, interquartile range.

### Association of morphologic and RECIST response with OS

**Figure 2:**
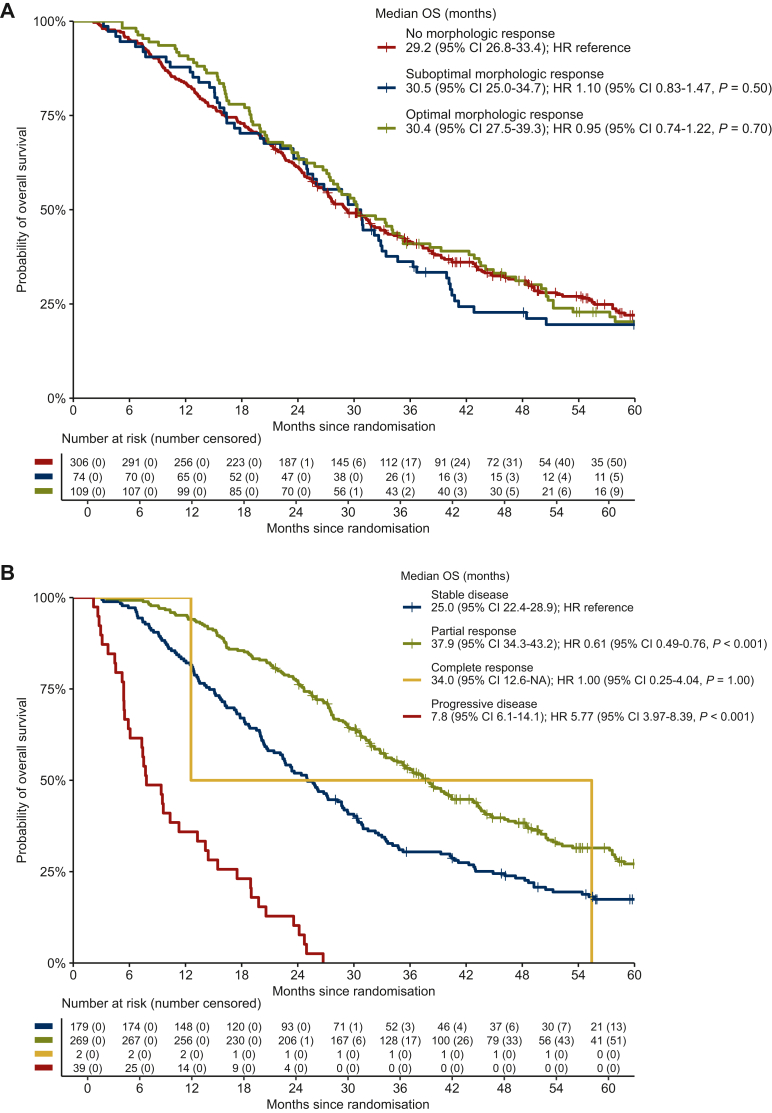
**Overall survival in all patients.** Overall survival according to (A) best morphologic response and (B) best RECIST response. CI, confidence interval; HR, hazard ratio; OS, overall survival.

Morphologic response was not significantly associated with OS (Figure 2A). Limiting this analysis to patients with RECIST-stable disease also showed no difference between either suboptimal (HR 1.05, 95% CI 0.66-1.64, *P* = 0.85) or optimal morphologic response (HR 0.87, 95% CI 0.60-1.26, *P* = 0.46) compared with no morphologic response. Similar results were observed in patients who underwent no local treatment with no differences for suboptimal (HR 1.07, 95% CI 0.73-1.59, *P* = 0.72) and optimal morphologic response (HR 0.78, 95% CI 0.53-1.16, *P* = 0.22) compared with no morphologic response. RECIST response was significantly associated with OS (Figure 2B). Outcomes were comparable for patients treated with bevacizumab-containing treatment (Supplementary Figure S1, available at https://doi.org/10.1016/j.esmoop.2024.104075).

### Association of morphologic and RECIST response with pathological response in patients who underwent local treatment

Pathological response is shown per morphologic response and RECIST category in Supplementary Figure S2, available at https://doi.org/10.1016/j.esmoop.2024.104075. Major pathological response was observed in 19 of 57 (33%) patients with optimal morphologic response compared with 47 of 185 (25%) patients with suboptimal or no morphologic response (*P* = 0.24). Regarding RECIST, 46 of 157 (29%) patients with complete or partial response had a major pathological response compared with 20 of 85 (24%) patients with stable, progressive, or non-evaluable disease (*P* = 0.34). Pathological response according to the percentage of viable tumour cells was also similar across morphologic response and RECIST categories (Supplementary Figure S3, available at https://doi.org/10.1016/j.esmoop.2024.104075). Comparable results were observed in patients receiving bevacizumab-containing treatment (Supplementary Figure S4, available at https://doi.org/10.1016/j.esmoop.2024.104075).

### Association of morphologic, RECIST, and pathological response with OS in patients who underwent local treatment

**Figure 3:**
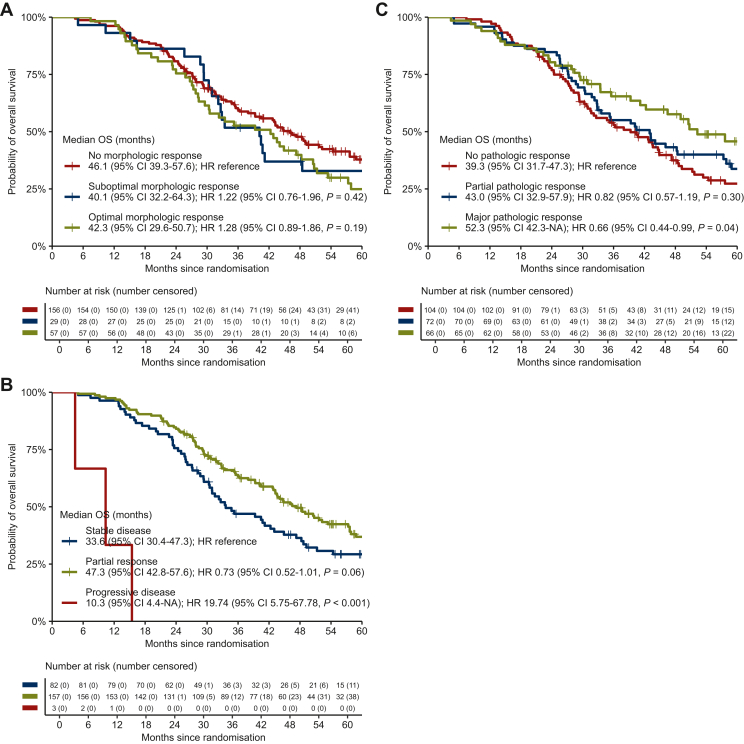
**Overall survival in patients who underwent local treatment.** Association of morphologic (A), RECIST (B), and pathological (C) response with overall survival in patients who underwent local treatment. CI, confidence interval; HR, hazard ratio; NA, not applicable; OS, overall survival.

Morphologic response was not associated with OS (Figure 3A). Three patients with RECIST progressive disease underwent local treatment, which was significantly associated with worse OS compared with stable disease (HR 19.74, 95% CI 5.75-67.78, *P* < 0.001; Figure 3B). OS was significantly better in patients with major pathological response compared with no pathological response (HR 0.66, 95% CI 0.44-0.99, *P* = 0.04; Figure 3C). OS was not significantly different according to pathological response based on the percentage of viable tumour cells (Supplementary Figure S5, available at https://doi.org/10.1016/j.esmoop.2024.104075). Similar results were observed in patients treated with bevacizumab-containing systemic treatment only (Supplementary Figure S6, available at https://doi.org/10.1016/j.esmoop.2024.104075).

### Association of morphologic, RECIST, and pathological response with early recurrence in patients who underwent complete local treatment

**Figure 4:**
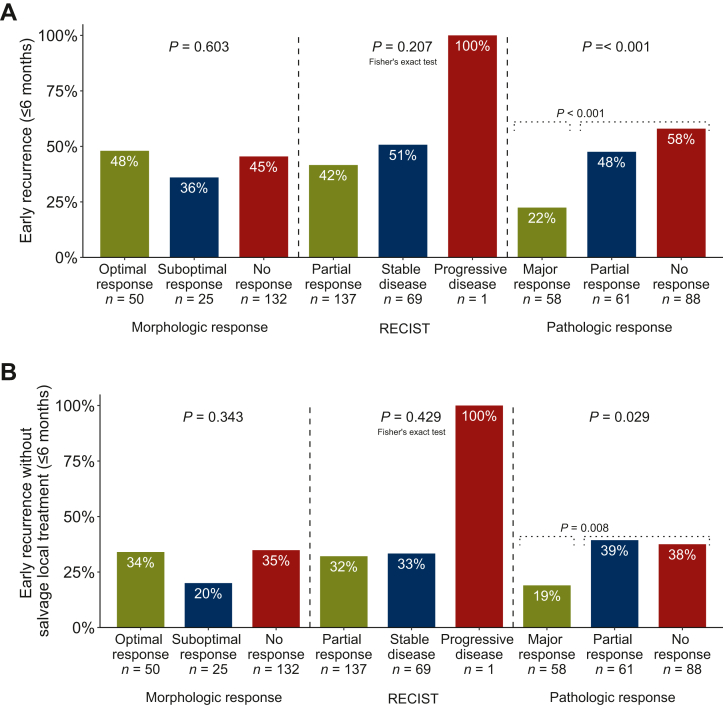
**Early recurrence in patients who underwent complete local treatment.** Association of morphologic, RECIST, and pathological response with early recurrence (A) and early recurrence without salvage local treatment (B) in patients who underwent complete local treatment.

Early recurrence was not different between patients with optimal, suboptimal, and no morphologic response [48% (24/50), 36% (9/25), and 45% (60/132), respectively, *P* = 0.60, Figure 4A], and neither was early recurrence without salvage local treatment [34% (17/50), 20% (5/25), and 35% (46/132), respectively, *P* = 0.34, Figure 4B]. No significant differences were observed between RECIST categories; early recurrence occurred in 57 of 137 (42%) patients with partial response, 35 of 69 (51%) patients with stable disease, and 1 of 1 (100%) patient with progressive disease (*P* = 0.25; Figure 4A), and early recurrence without salvage local treatment occurred in 44 of 137 (32%) patients, 23 of 69 (33%) patients, and 1 of 1 (100%) patient (*P* = 0.43; Figure 4B), respectively. Pathological response was significantly associated with early recurrence (*P* < 0.001) which occurred in 13 of 58 (22%) patients with major response, 29 of 61 (48%) patients with partial response, and 51 of 88 (58%) patients with no response (Figure 4A). This effect was mainly caused by the difference in patients with major compared with no or partial response; early recurrence occurred in 13 of 58 (22%) patients with major response and 80 of 149 (54%) patients with no or partial response (*P* < 0.001; Figure 4A). Early recurrence without salvage local treatment was also significantly different between major response [19% (11/58)], partial response [39% (24/61)], and no response [38% (33/88); *P* = 0.029; Figure 4B]. Early recurrence without salvage local treatment was more often observed in patients with either no or partial pathological response [38% (57/149)] compared with patients with major pathological response [19% (11/58); *P* = 0.029; Figure 4B]. Comparable results were found for pathological response based on the percentage of viable tumour cells (Supplementary Figure S7, available at https://doi.org/10.1016/j.esmoop.2024.104075). Results for patients treated with bevacizumab-containing treatment were also similar (Supplementary Figure S8, available at https://doi.org/10.1016/j.esmoop.2024.104075).

### Association of pathological characteristics of CRLM with OS in patients who underwent local treatment

The association between pathological characteristics and OS in patients who underwent local treatment is shown in Supplementary Table S2, available at https://doi.org/10.1016/j.esmoop.2024.104075. Compared with a desmoplastic histopathological growth pattern, non-desmoplastic is associated with worse OS (HR 1.68, 95% CI 1.20-2.37, *P* = 0.003). A higher proportion of fibrosis was associated with better OS (HR 0.99, 95% CI 0.99-1.00, *P* = 0.023).

## Discussion

In this study with patients with initially unresectable CRLM, morphologic response was not prognostic for early recurrence, early recurrence without salvage local treatment, and OS. Morphologic response was not associated with pathological response either. The early recurrence outcomes occurred less frequently in patients with major pathological response, and OS was significantly longer in this group compared with patients with no or partial pathological response. Since pathological response is only available after surgery, this cannot be used to avoid potentially futile liver surgery.

Our results confirm the prognostic value of RECIST in patients with metastatic colorectal cancer.^24^ In patients who underwent local treatment, OS was numerically but not significantly longer in patients with partial response versus stable disease and was significantly worse in three patients with progressive disease. Previous studies have reported no differences in OS between RECIST categories, except for one study which found worse OS in patients with progressive disease, following neoadjuvant chemotherapy in patients with upfront resectable CRLM.^25^, ^26^, ^27^ RECIST was not associated with early recurrence outcomes. A potential explanation for this inconsistency is that a strong response to systemic treatment suggests that the tumour possesses characteristics making it sensitive to both the initial and subsequent lines of treatment. This sensitivity to systemic treatment provides a rationale for the observed association with OS, as well as for the absence of an association with early recurrence.

Previous studies have shown variable results on the prognostic value of morphologic response for OS. The studies with positive results were all carried out in patients who underwent resection after preoperative fluoropyrimidine-based chemotherapy with or without bevacizumab.^3^, ^4^, ^5^ The prognostic value of morphologic response for OS was not observed in other studies in this population, nor in patients with unresectable CRLM or receiving anti-EGFR-containing treatment.^6^, ^7^, ^8^, ^9^, ^10^ There is no clear explanation for the absence of external validity, although the fact that morphologic response assessment is subject to considerable interobserver variability may play a role.^28^

Pathological response is a well-known surrogate marker for OS that can be assessed by the TRG criteria^19^ or the percentage of viable tumour cells^20^ in case of CRLM. TRG appeared to be the strongest prognostic determinant that stratified patients into different risk groups for OS.^29^ As with all manual response measurements, interobserver variability may have contributed to the results found in our study where major but not partial response was better than no pathological response. Nevertheless, the differences in study populations may have played a greater role. Patients in our study had more extensive disease, expressed by the number and size of CRLM, compared with previous studies evaluating the association between TRG and OS. This higher burden of disease may have overruled the prognostic value of pathological response. However, our data confirm that a desmoplastic histopathological growth pattern is associated with a better OS.^30^^,^^31^ Since all patients received preoperative systemic treatment, we could not determine whether the desmoplastic growth pattern was present initially or developed as a result of the treatment.

A strength of this study is that we confirmed our main results in subgroup analyses, which only included patients receiving bevacizumab-based treatment and patients who received local treatment, since the morphologic response criteria were developed in this population.^3^ Some patients could have been treated locally upfront by adding ablation or by carrying out a two-stage resection with or without portal vein ablation due to our baseline unresectability criterion (unresectable if an R0 resection could not be achieved with surgical resection only in one stage). This could imply that patients with upfront technically resectable disease are overrepresented in the RECIST-stable disease group. This might have led to an overestimation of OS in patients with stable disease, potentially resulting in a lack of prognostic value for RECIST in patients with local treatment. Another limitation of our study is that we have condensed the morphologic and pathological responses of multiple metastases from the same patient into a single category without considering tumour heterogeneity. This pragmatic approach was also used in previously published studies.

In conclusion, our results indicate that morphologic response cannot replace RECIST in patients with initially unresectable CRLM. For patients eligible for local treatment, morphologic and RECIST response are not associated with early recurrence. Novel preoperative parameters are warranted to predict early recurrence and prevent potentially futile liver surgery.
