# Supplementary material for: Prognostic value of radiologic and pathological response in colorectal cancer liver metastases upon systemic induction treatment: subgroup analysis of the CAIRO5 trial
Source: ESMO Open. 2024 Dec 11;9(12):104075. doi: 10.1016/j.esmoop.2024.104075 (PMC11697041; doi:10.1016/j.esmoop.2024.104075)
Supplement: Supplementary Material [file mmc1.docx]

**Supplementary tables and figures**

**Supplementary Table 1** Morphologic response per treatment group

|  | | Right-sided and/or *RAS* or *BRAF^V600E^* mutated tumour | | | | | Left-sided and *RAS* and *BRAF^V600E^* wild-type tumour | | | | |
| --- | --- | --- | --- | --- | --- | --- | --- | --- | --- | --- | --- |
|  | FOLFOX/  FOLFIRI plus bevacizumab (n=141) | | | FOLFOXIRI plus bevacizumab (n=133) | | FOLFOX/  FOLFIRI plus bevacizumab (n=106) | | | | FOLFOX/  FOLFIRI plus panitumumab (n=109) | |
| Best morphologic response |  | | |  | |  | | | |  | |
| No response | 90 (64%) | | | 71 (53%) | | 64 (60%) | | | | 81 (74%) | |
| Suboptimal response | 19 (13%) | | | 22 (17%) | | 19 (18%) | | | | 14 (13%) | |
| Optimal response | 32 (23%) | | | 40 (30%) | | 23 (22%) | | | | 14 (13%) | |
| Best RECIST response |  | | |  | |  | | | |  | |
| Stable disease | 67 (48%) | | | 53 (40%) | | 43 (41%) | | | | 16 (15%) | |
| Partial response | 48 (34%) | | | 75 (56%) | | 57 (54%) | | | | 89 (82%) | |
| Complete response | 0 (0%) | | | 1 (1%) | | 0 (0%) | | | | 1 (1%) | |
| Progressive disease | 26 (18%) | | | 4 (3%) | | 6 (6%) | | | | 3 (3%) | |
|  |  | |  | |  | | |  |  | |  |

**Supplementary Figure 1** Overall survival according to A) best morphologic response and B) best RECIST response in patients receiving bevacizumab-containing treatment.

A

**
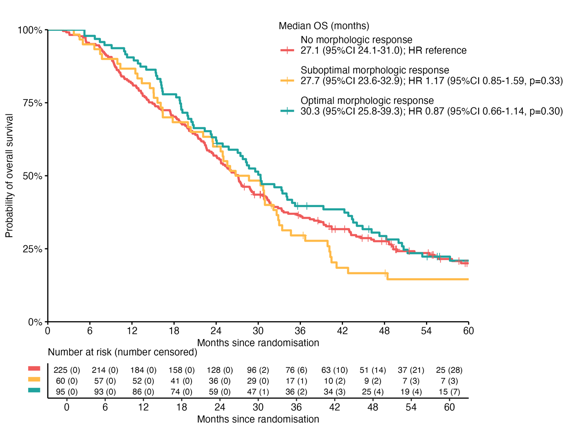
**

B

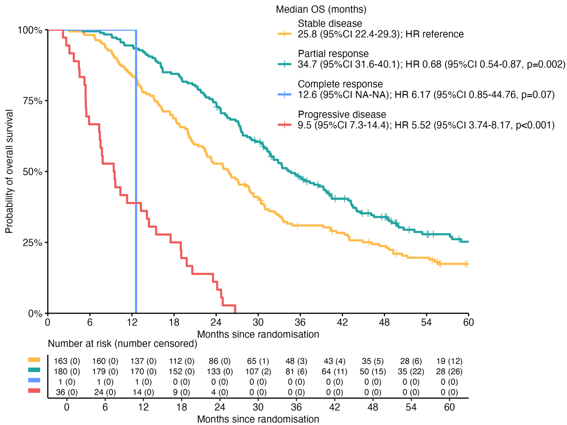


**Supplementary Figure 2** Association of morphologic and RECIST response with pathologic response according to the TRG criteria in patients who underwent local treatment


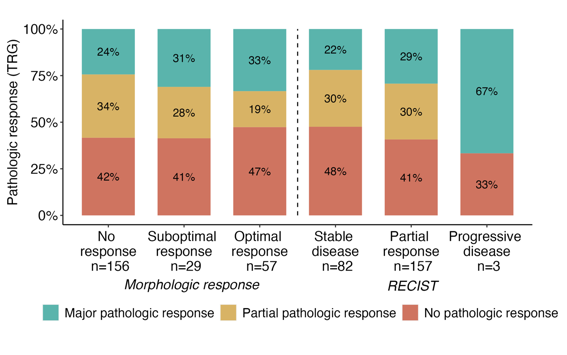


**Supplementary Figure 3** Association of morphologic and RECIST response with pathologic response according to the percentage of viable tumour cells (Blazer et al.) in patients who underwent local treatment.


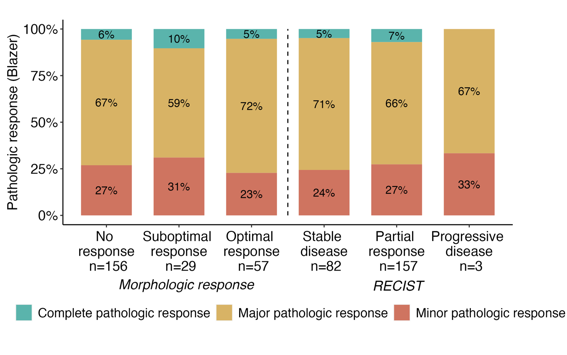


**Supplementary Figure 4** Association of morphologic and RECIST response with pathologic response in patients who underwent local treatment receiving bevacizumab-containing systemic treatment. Pathologic response according to the TRG criteria (A) and percentage of viable tumour cells (Blazer et al.; B)

A

**
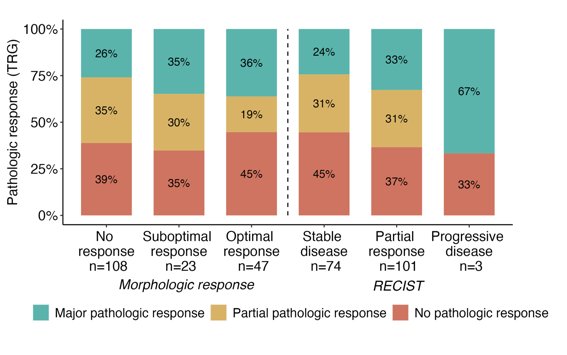
**

B

**
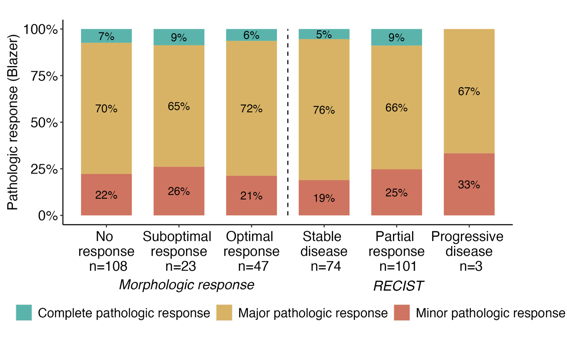
**

**Supplementary Figure 5** Association of pathologic response based on the percentage of viable tumour cells (Blazer et al.) with overall survival in patients who underwent local treatment.

**
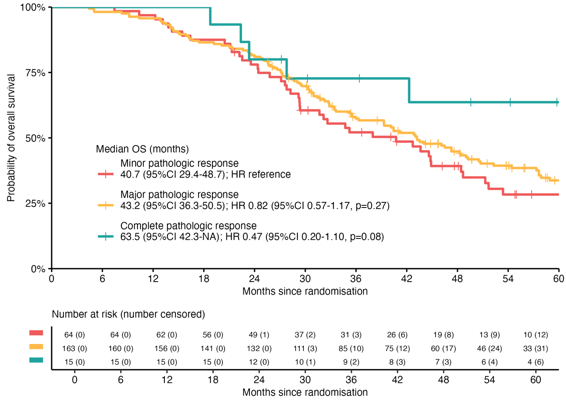
**

**Supplementary Figure 6** Association of morphologic (A), RECIST (B), and pathologic (C) response with overall survival in patients who underwent local treatment receiving bevacizumab-containing systemic treatment.


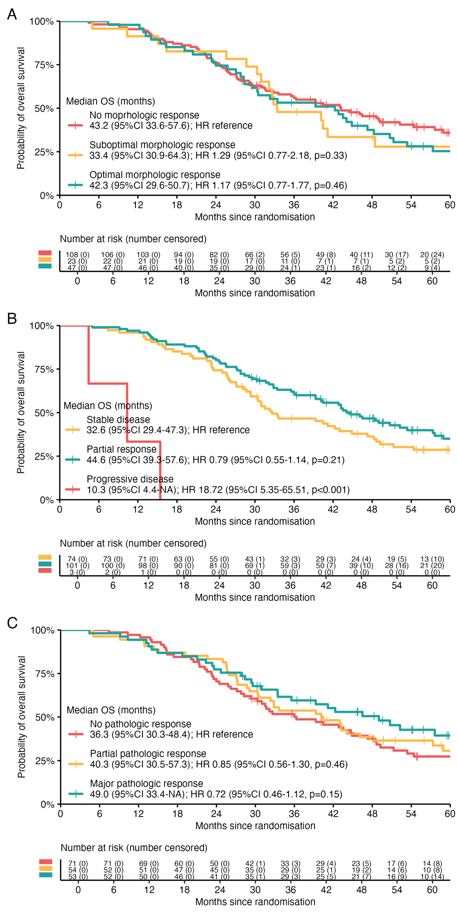


**Supplementary Figure 7** Association of pathologic response according to Blazer et al. based on percentage of viable tumour cells with early recurrence (A) and early recurrence without salvage local treatment (B) in patients who underwent complete local treatment. Fisher’s exact test was used for all comparisons.

A)


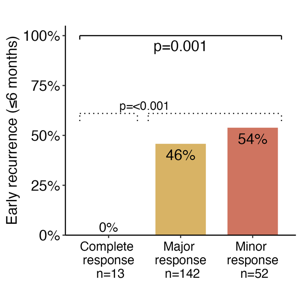


B)


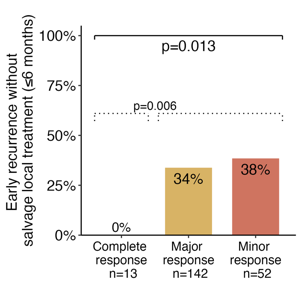


**Supplementary Figure 8** Association of morphologic, RECIST, and pathologic response with early recurrence (A) and early recurrence without salvage local treatment (B) in patients who underwent complete local treatment receiving bevacizumab-containing treatment.

A


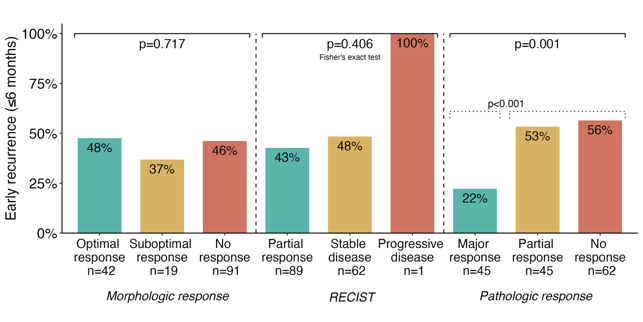


B

**
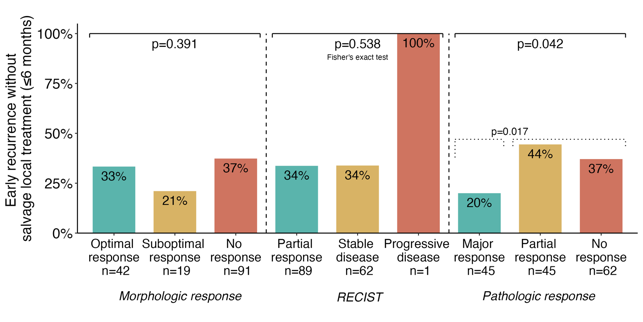
**

**Supplementary Table 2** Association of pathologic characteristics with OS in patients who underwent local treatment

| ****Characteristic**** | ****Event Rate**** | ****HR*****^1^* | ****95% CI*****^1^* | ****p-value**** | |
| --- | --- | --- | --- | --- | --- |
| Desmoplastic vs non-desmoplastic HGP^1^ |  |  |  |  | |
| Desmoplastic | 55 / 99 (56%) | — | — |  | |
| Non-desmoplastic | 86 / 117 (74%) | 1.68 | 1.20, 2.37 | 0.003 | |
| Unknown | 12 / 26 (46%) | 0.76 | 0.41, 1.43 | 0.398 | |
| Predominant HGP^1^ |  |  |  |  | |
| Desmoplastic HGP | 55 / 99 (56%) | — | — |  | |
| Mixed HGP (all HGP ≤ 50%) | 29 / 39 (74%) | 1.89 | 1.20, 2.97 | 0.006 | |
| Pushing HGP | 2 / 2 (100%) | 2.02 | 0.49, 8.31 | 0.329 | |
| Replacement HGP | 55 / 75 (73%) | 1.61 | 1.11, 2.35 | 0.012 | |
| Sinusoidal HGP | 0 / 1 (0%) | — | — | — | |
| Unknown | 12 / 26 (46%) | 0.76 | 0.41, 1.43 | 0.398 | |
| Peritumoral lymphocytes |  |  |  |  | |
| No | 24 / 34 (71%) | — | — |  | |
| Sporadically or more than sporadically | 129 / 208 (62%) | 0.69 | 0.44, 1.06 | 0.090 | |
| Portal lymphocytes |  |  |  |  | |
| No | 105 / 166 (63%) | — | — |  | |
| Yes | 48 / 74 (65%) | 0.79 | 0.56, 1.11 | 0.170 | |
| Unknown | 0 / 2 (0%) | — | — | — | |
| Mean % tumour cells | 153 / 242 (63%) | 1.00 | 1.00, 1.01 | 0.122 | |
| Mean % necrosis | 153 / 242 (63%) | 1.00 | 0.99, 1.01 | 0.819 | |
| Mean % fibrosis | 153 / 242 (63%) | 0.99 | 0.99, 1.00 | 0.023 | |
| Mean % mucin | 153 / 242 (63%) | 1.00 | 0.99, 1.01 | 0.776 | |
| Tumour budding |  |  |  |  | |
| No | 135 / 216 (62%) | — | — |  | |
| Yes | 13 / 16 (81%) | 1.44 | 0.82, 2.55 | 0.207 | |
| Unknown | 5 / 10 (50%) | 0.55 | 0.22, 1.34 | 0.186 | |
| Mucinous pattern |  |  |  |  | |
| No | 142 / 224 (63%) | — | — |  | |
| Yes | 11 / 18 (61%) | 0.99 | 0.53, 1.82 | 0.964 | |
| *^1^* HR = Hazard Ratio, CI = Confidence Interval, HGP = histological growth pattern | | | | |  |
